# Exploring the relationship between anal fistula and colorectal cancer based on Mendelian randomization and bioinformatics

**DOI:** 10.1111/jcmm.18537

**Published:** 2024-08-09

**Authors:** Yicheng Liu, Wenjun Zhao, Weiye Hu, Jin Xu, Haiyan Zhang, Ting Huang, Chuang Wu, Jiajia Yang, Wenjing Mao, Xiaobing Yao, Yafeng Lu, Qingming Wang

**Affiliations:** ^1^ Department of Anorectal Surgery Shanghai Baoshan Hospital of Intergrated Traditonal Chinese and Western Medicine Shanghai China; ^2^ Department of Liver, Yueyang Hospital of Integrated Traditional Chinese and Western Medicine Shanghai University of Traditional Chinese Medicine Shanghai China; ^3^ Department of Gastrointestinal Surgery, Yueyang Hospital of Integrated Traditional Chinese and Western Medicine Shanghai University of Traditional Chinese Medicine Shanghai China; ^4^ Department of Anorectal Surgery, Shuguang Hospital Shanghai University of Traditional Chinese Medicine Shanghai China

**Keywords:** anal fistula, colorectal cancer, core genes, machine learning, prognostic analysis

## Abstract

The association between anal fistula patients and colorectal cancer, as well as the potential pathophysiological mechanisms, remains unclear. To explore the relationship between anal fistula and colorectal cancer and its potential mechanisms. Analysis of GEO and TCGA databases. Disease‐related genes were also referenced from Coremine Medical, GeneCard and OMIM. Core hub genes were identified through protein–protein interaction analysis by intersecting differentially expressed genes from the datasets with disease data. On one hand, a prognostic model was developed using genes and its prognostic role was validated. On the other hand, the optimal diagnostic genes were selected through machine learning. Mendelian randomization (MR) analysis was conducted to explore the potential causal link between anal fistula and colorectal cancer. Thirteen core genes were identified (TMEM121B, PDGFRA, MID2, WNT10B, HOXD13, BARX1, SIX2, MMP1, SNAL1, CDKN2A, ITGB3, TIMP1, CALB2). Functional enrichment analysis revealed that the intersecting genes between anal fistula and colorectal cancer were associated with extracellular matrix components, signalling pathways, cell growth, protein modification, as well as important roles in cellular activities, tissue and organ development, and biological function maintenance. These genes were also involved in pathways related to Wnt signalling and colorectal cancer development. Prognostic analysis and immune infiltration analysis indicated a close relationship between core hub genes and the prognosis and immune infiltration in colorectal cancer. Machine learning showed that core genes played an essential role in the diagnostic differentiation of colorectal cancer. MR results suggested no causal relationship between anal fistula and colorectal cancer. This study identified shared core genes between anal fistula and colorectal cancer, involved in various pathways related to tumour development. These genes play crucial roles in prognosis and diagnosis.

## 1 INTRODUCTION

Colorectal cancer (CRC) stands out as a malignancy with elevated incidence and mortality rates globally, presenting a significant challenge to public health.^1^ The prevalence of colorectal cancer has been on the incline in recent years due to lifestyle modifications and the aging population.^2^, ^3^ Hence, conducting comprehensive research on the pathogenesis, risk factors and preventive strategies for colorectal cancer holds immense importance in mitigating its occurrence and enhancing the well‐being of affected individuals.

Anal fistula, as a type of anorectal disease, refers to an abnormal channel formed between the anal canal or rectum and the skin or other surrounding tissues.^4^ However, current research on the association between anal fistulas and colorectal cancer is insufficient, and their potential pathophysiological mechanisms are not yet clear. This study aims to explore whether anal fistula patients have a higher risk of developing colorectal cancer and to elucidate the possible mechanisms between the two.

This study will combine bioinformatics analysis to explore the potential connection between anal fistulas and colorectal cancer at the molecular level. By integrating genetics and bioinformatics data and methods, this study aims to provide new perspectives and strategies for the prevention and treatment of colorectal cancer, this study adopts Mendelian randomization (MR) method to assess causality through the natural randomization of genetic mechanisms, thereby providing more reliable causal inferences.^5^

## 2 MATERIALS AND METHODS

### 2.1 Datasets and collection

GSE134834 includes the detection of differences in LncRNA/mRNA expression between five pairs of colorectal tumour tissues and normal tissues. Additionally, disease‐related genes were sourced from Coremine Medical, GeneCard, and OMIM. Colorectal tissue data from GTEx (http://commonfund.nih.gov/GTEx/) was used as input for diagnostic machine learning. The exposure and outcome of MR were IEU‐b‐4965 (including 5657 cases and 372,016 controls) and UKB‐b‐6721 (including 1003 cases and 462,007 controls) from IEU OpenGWAS, both from European populations.

### 2.2 Selection of core hub genes

First, the disease‐related genes intersection was identified, and a Venn diagram was generated. Subsequently, the protein–protein interaction network analysis of these intersecting genes was carried out using the STRING website, and the outcomes were imported into Cytoscape for further examination, which led to the identification of six central hub genes. Finally, a functional enrichment analysis of these core hub genes was performed. Functional annotation of the core hub gene set was performed using the Metascape database.^6^

### 2.3 Unsupervised clustering

Unsupervised clustering analysis was conducted to categorize the samples. The R package ‘ConsensusClusterPlus’ (v1.68.0) was utilized for 1000 iterations to validate the reliability of the classification. This clustering algorithm was employed to ascertain both the quantity and robustness of the clusters.^7^

### 2.4 Establishment of the model

Based on the survival status and survival time of patients, the differentially expressed genes related to prognosis are analysed using unsupervised clustering methods to classify patients into several groups for further analysis. Subsequently, a scoring system is constructed using PCA (principal component analysis) method by selecting components 1 and 2 for this scoring system.^8^

### 2.5 Clinical functional assessment

To comprehend the correlation between model scores and clinical characteristics, we examined the association between model scores and individual patient attributes including gender, age, stage, pathology and survival status. Additionally, we confirmed the link between model scores and the survival rates pertaining to various independent clinical features. Univariate and multivariate Cox analyses were conducted to investigate the relationship between model scores and survival rates.^9^

### 2.6 Immune infiltration analysis

After grouping the main variables, the data were subjected to corresponding statistical analysis to obtain the distribution of each group within each category.^10^ The statistical data was visualized using the ggplot2 package to create overlaid bar charts. Based on the core algorithm of CIBERSORT (CIBERSORT.R script analysis), markers for 22 immune cells provided by the CIBERSORTx website (https://cibersortx.stanford.edu/) were used to calculate the immune infiltration status of the uploaded data^11^, ^12^. The stromal and immune scores of colorectal cancer patients from TCGA were calculated using the R package ‘ESTIMATE’ (v1.0.13).^13^

### 2.7 Machine learning

Extracting colorectal cancer corresponding TCGA data and matched normal tissue data from GTEx, split at a 1:1:1 ratio into training set (Dataset A) and three test sets (Dataset B, Dataset C), as well as an internal validation set (Dataset D) randomly sampled from the first three sets. Fifteen machine learning algorithms are employed, including neural network, Lasso regression, and naive Bayes and so forth. For each model, the C‐index is calculated on test sets 1, 2, 3, and the internal validation set. Models are then ranked based on average C‐index, AUC area, recall, and F‐value.^14^ A 10‐fold cross‐validation framework was applied to evaluate the performance of multiple machine learning models.

### 2.8 Mendelian randomization

In our analysis, we extracted phenotype‐related SNPs (*p* < 5 × 10^−8^). The impact of palindromic SNPs was also considered. In order to enhance the robustness and reliability of the conclusions, a variety of rigorous analysis methods were employed in this study. These methods include inverse variance weighting, MR‐PRESSO, modulus‐based estimation, weighted median, MR‐Egger regression, and MR‐RAPS. These approaches incorporate different assumptions in the two‐sample MR analysis.^15^, ^16^, ^17^ The genetic variants' level of pleiotropy, heterogeneity, and stability on bone mineral density (BMD) were evaluated through MR‐Egger intercept test.^18^, ^19^, ^20^ Anomalies identified through the MR‐PRESSO outlier test were systematically eliminated to mitigate the influence of heterogeneity and pleiotropy.^19^, ^21^

### 2.9 Statistics

All statistical analyses were conducted using R version 4.2.1 and the TwoSampleMR (v0.6.4) package. A *p*‐value <0.05 was considered statistically significant.^22^

## 3 RESULTS

### 3.1 Screening of core hub genes for anal fistula and colorectal cancer

**FIGURE 1:**
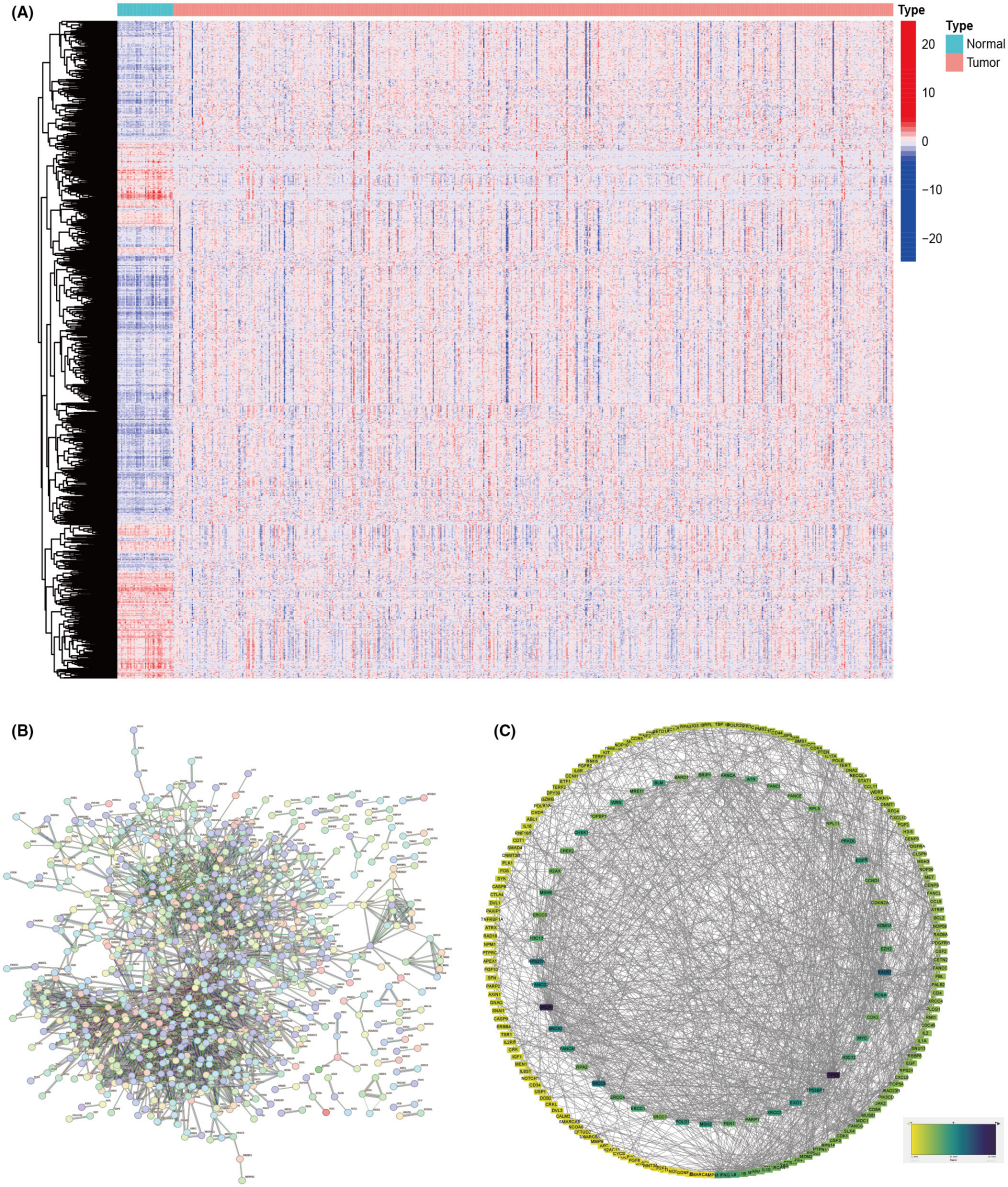
The core hub genes of anal fistula and colorectal cancer. (A) Venn diagram of anal fistula and colorectal cancer. (B) Protein interaction network of intersecting genes. (C) Core hub gene interaction network.

Genes related to anal fistula and colorectal cancer intersect with differentially expressed genes in colorectal cancer obtained from GSE134834 in disease databases, resulting in 14 intersecting genes (Figure 1A). Protein–protein interaction analysis was conducted on the STRING website (confidence = 0.04), and based on this, core hub genes were selected in the Cytoscape software (using the cytoHubba plugin), resulting in a total of six core hub genes (Figure 1B,C).

### 3.2 Functional Enrichment Analysis

**FIGURE 2:**
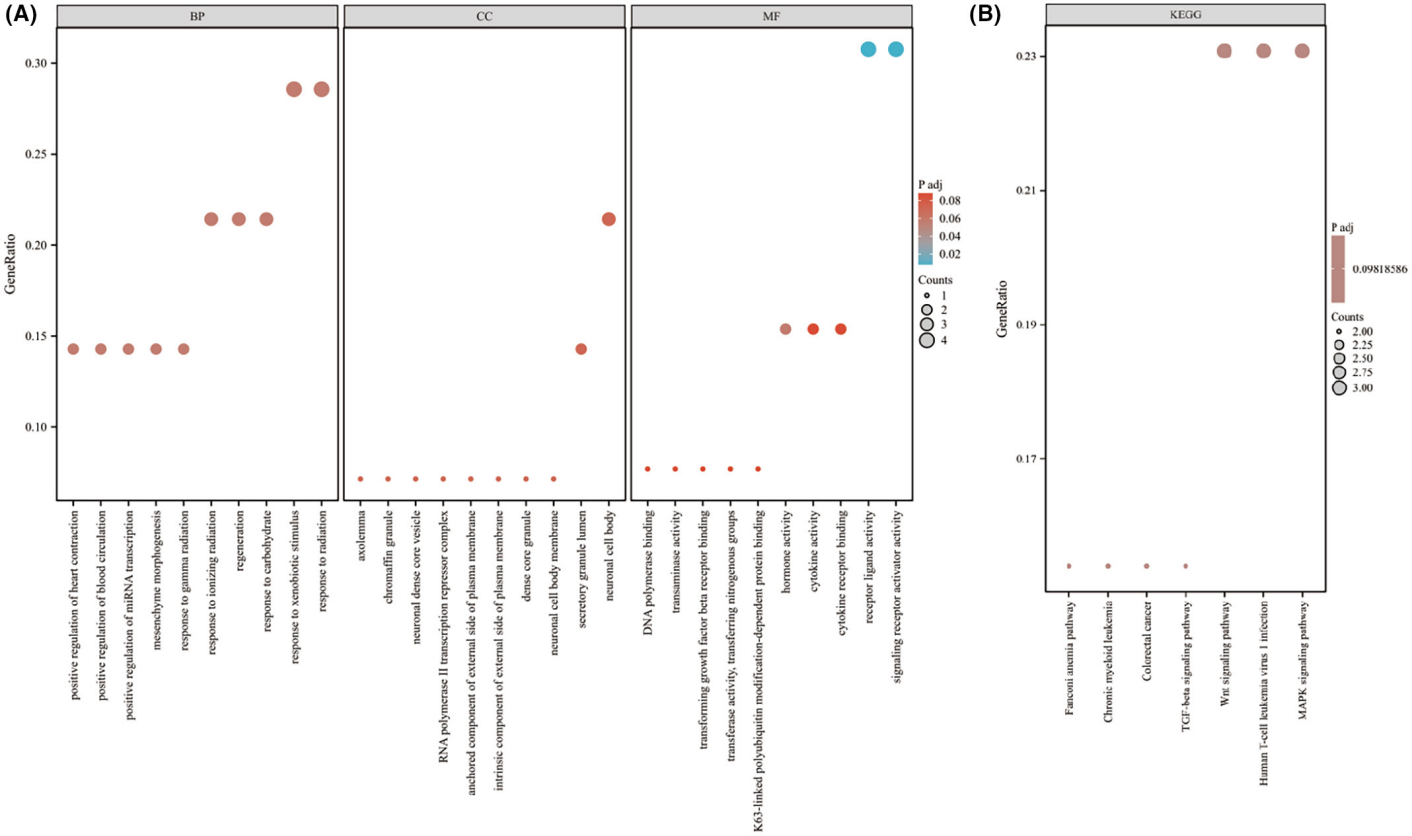
Functional enrichment. (A) GO enrichment. (B) KEGG enrichment.

Cell components are mainly divided into membrane‐related components, cytoplasm‐related components, and extracellular matrix‐related components. Extracellular matrix‐related components include extracellular matrix containing collagen, platelet alpha granules and lumens. Molecular functions mainly involve signal transduction, cell growth and protein modification. Biological functions cover the adaptation and regulation of organisms to different environmental factors and physiological processes, involving molecular level regulation, cell activities, tissue and organ development and function maintenance across multiple levels (Figure 2A). Pathways mainly include Wnt signalling pathway, Fanconi anaemia pathway, human T‐cell leukaemia virus infection, chronic myeloid leukaemia, colorectal cancer, TGF‐β signalling pathway, MAPK signalling pathway, etc. (Figure 2B).

### 3.3 Classification of colorectal cancer based on anal fistula‐related genes

**FIGURE 3:**
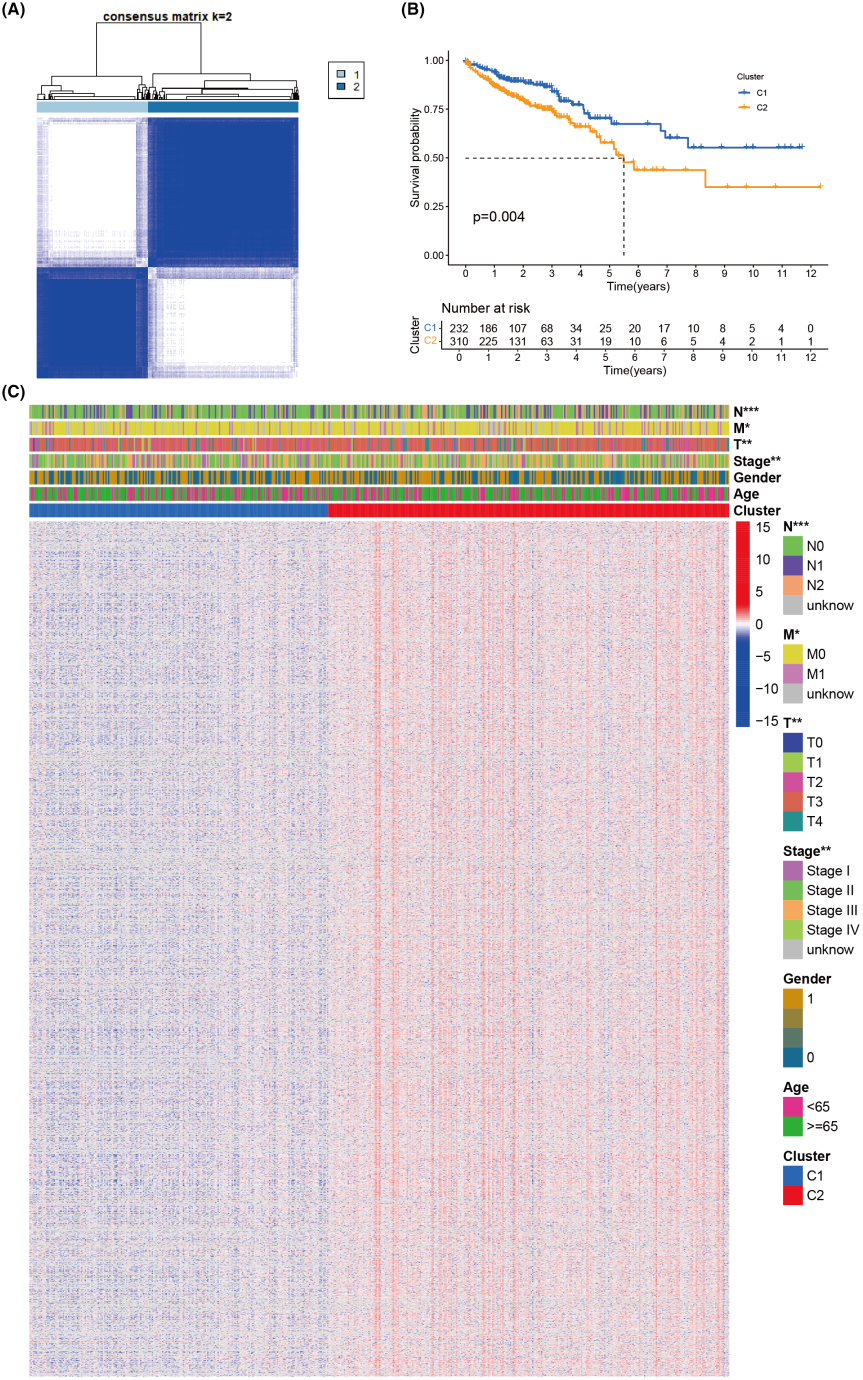
Colorectal cancer (CRC) typing based on anal fistula‐related genes. (A) Represents the typing display. (B) Represents the survival KM curve. (C) Represents the clinical information heatmap.

Figure 3A displays gene sequences, patient clinical information, anal fistula experimental results and colorectal cancer data. Figure 3B shows the difference in survival rates between patients with anal fistula combined with colorectal cancer and those with solitary colorectal cancer. The 5‐year survival rate of the combined group is lower compared to solitary colorectal cancer. Figure 3C shows the differences in tumour staging between the two groups of patients, with no differences in gender and age.

### 3.4 Clinical Functional Assessment

**FIGURE 4:**
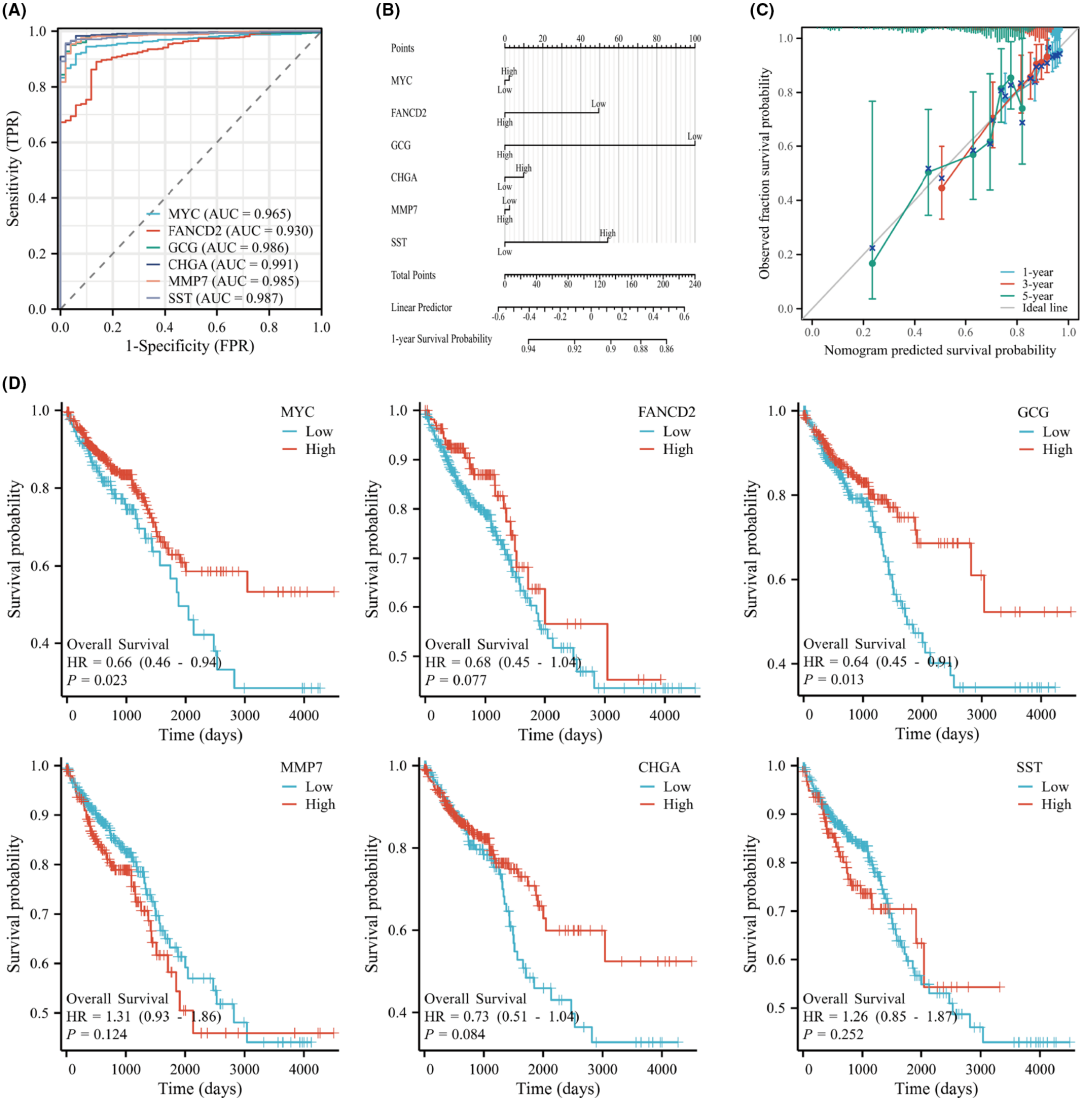
Clinical function evaluation of core hub genes. (A) Time‐dependent ROC. (B) Column chart. (C) Prognosis calibration. (D) Survival curve (KM plot).

The time‐dependent ROC, column chart, prognosis calibration and survival curve analyses were performed for the core hub genes. The results indicate that the overall predictive function of the core hub is good, and the individual predictive abilities of MYC and CGC are also very good. Figure 4.

### 3.5 Immune infiltration analysis

**FIGURE 5:**
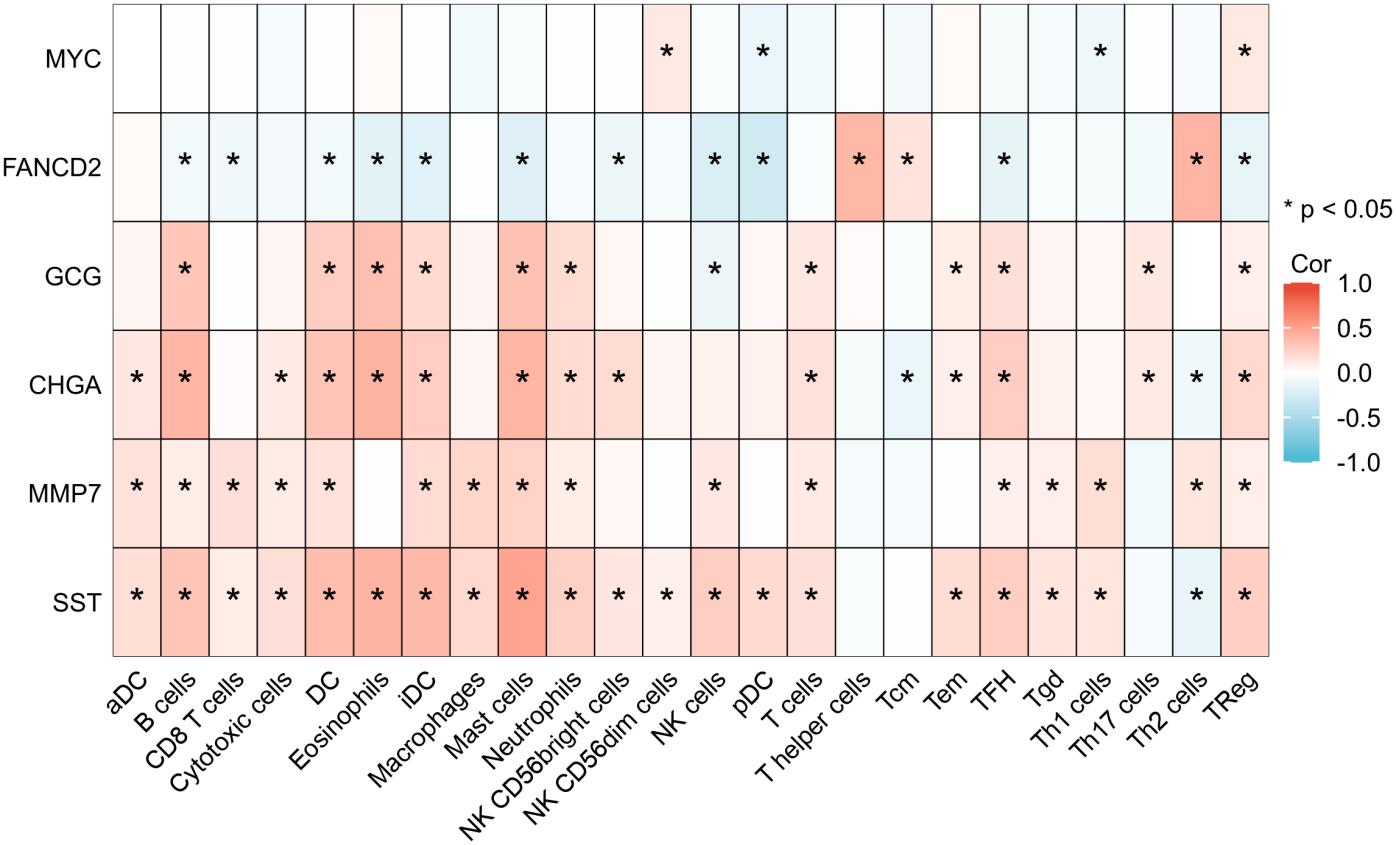
Immune‐related heatmap.

Based on the GSE134834 dataset, analyse the correlation between core hub genes and immune cells in colorectal cancer and generate an immune cell correlation heatmap (Figure 5). The heatmap results indicate that core hub genes are closely related to various immune cells in colorectal cancer, with a predominantly positive correlation.

### 3.6 Mutation of core hub genes in colorectal cancer

**FIGURE 6:**
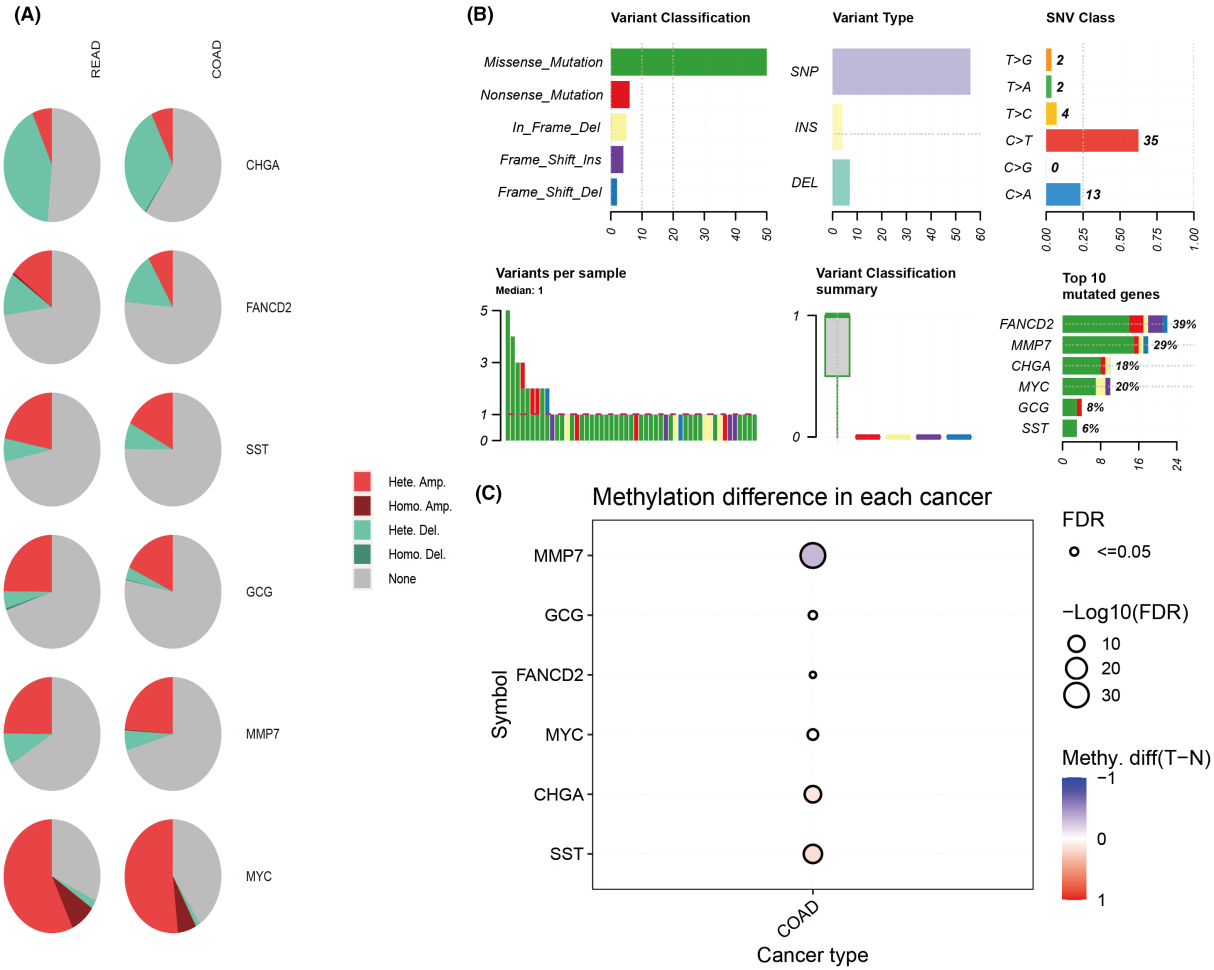
Mutation of hub genes in colorectal cancer (A) CNV (copy number variation). (B) SNV (single nucleotide variation). (C) Methylation. The number of normal samples with methylation data in READ is less than 10, so analysis cannot be performed.

The analysis of the mutations of core hub genes in colorectal cancer is based on the GSCA website. Copy number variations of core hub genes are widely present in colon and rectal cancers, with *CHGA* and *FANCD2* primarily showing copy number heterozygous deletion, while the rest mainly exhibit copy number heterozygous amplification (Figure 6A). The analysis of single nucleotide variations shows that core hub genes primarily have missense mutations in single nucleotide variations, with *FANCD2* and *MMP7* mutations being the most prevalent (Figure 6B). Although there is a lack of comparative data on the methylation of core hub genes in rectal cancer, there are methylation differences of core hub genes in colon cancer (Figure 6C).

### 3.7 The drug network and ceRNA network of core hub genes

**FIGURE 7:**
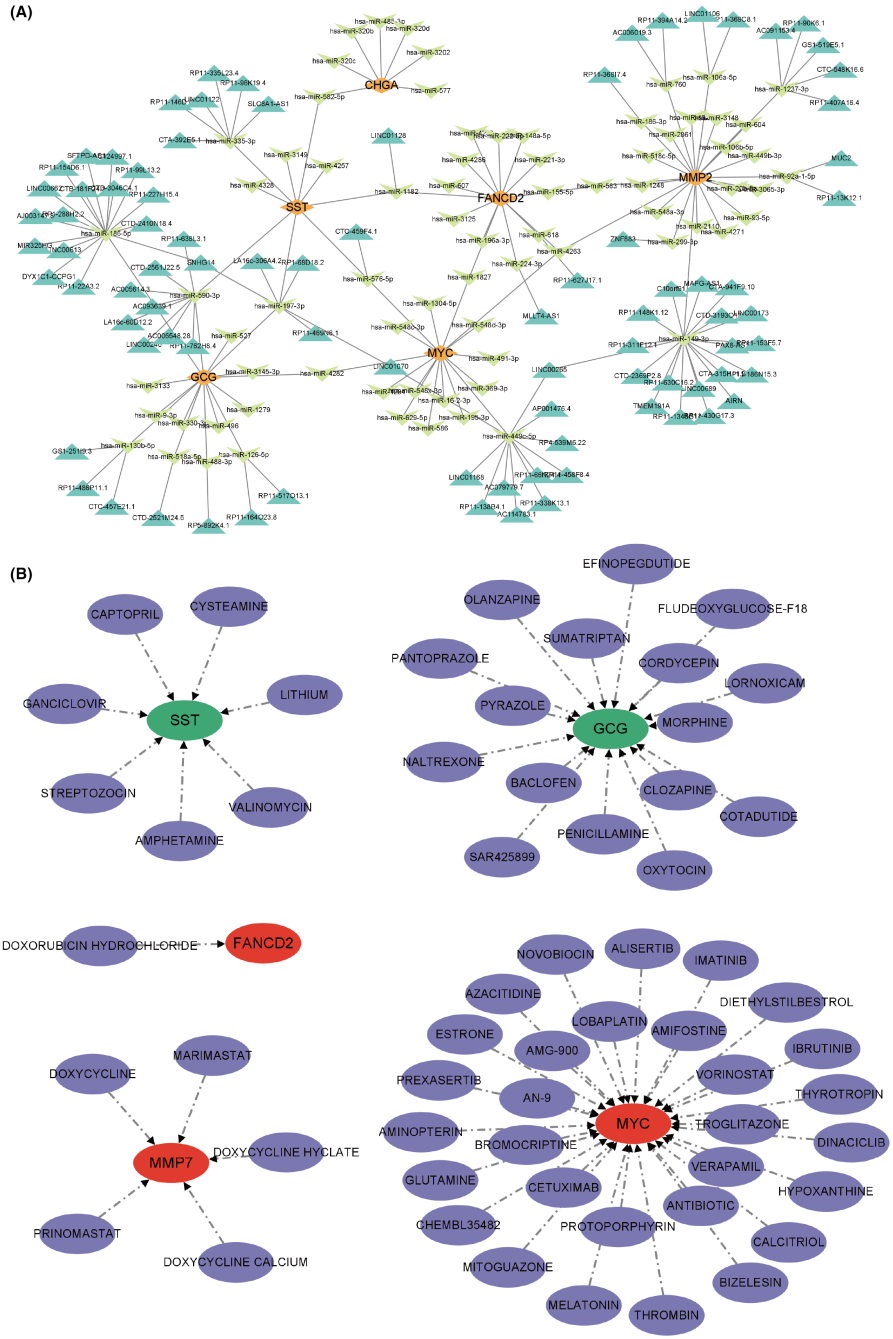
Drug network and ceRNA network. (A) ceRNA network. (B) Drug network. Green represents genes downregulated in colorectal cancer, while red represents genes upregulated in colorectal cancer.

The ceRNA network of core hub genes was constructed based on miRanda, miRDB, TargetScan and spongeScan. The results indicate that the ceRNA network between core hub genes is intricate, suggesting that besides protein interactions, core hub genes can also be regulated by complex non‐coding RNAs (Figure 7A). The drug network of core hub genes was drawn based on DGIDB, and except for *CHGA*, all other core genes have drugs targeting them (Figure 7B).

### 3.8 The protein expression levels of six core hub genes in tissues

**FIGURE 8:**
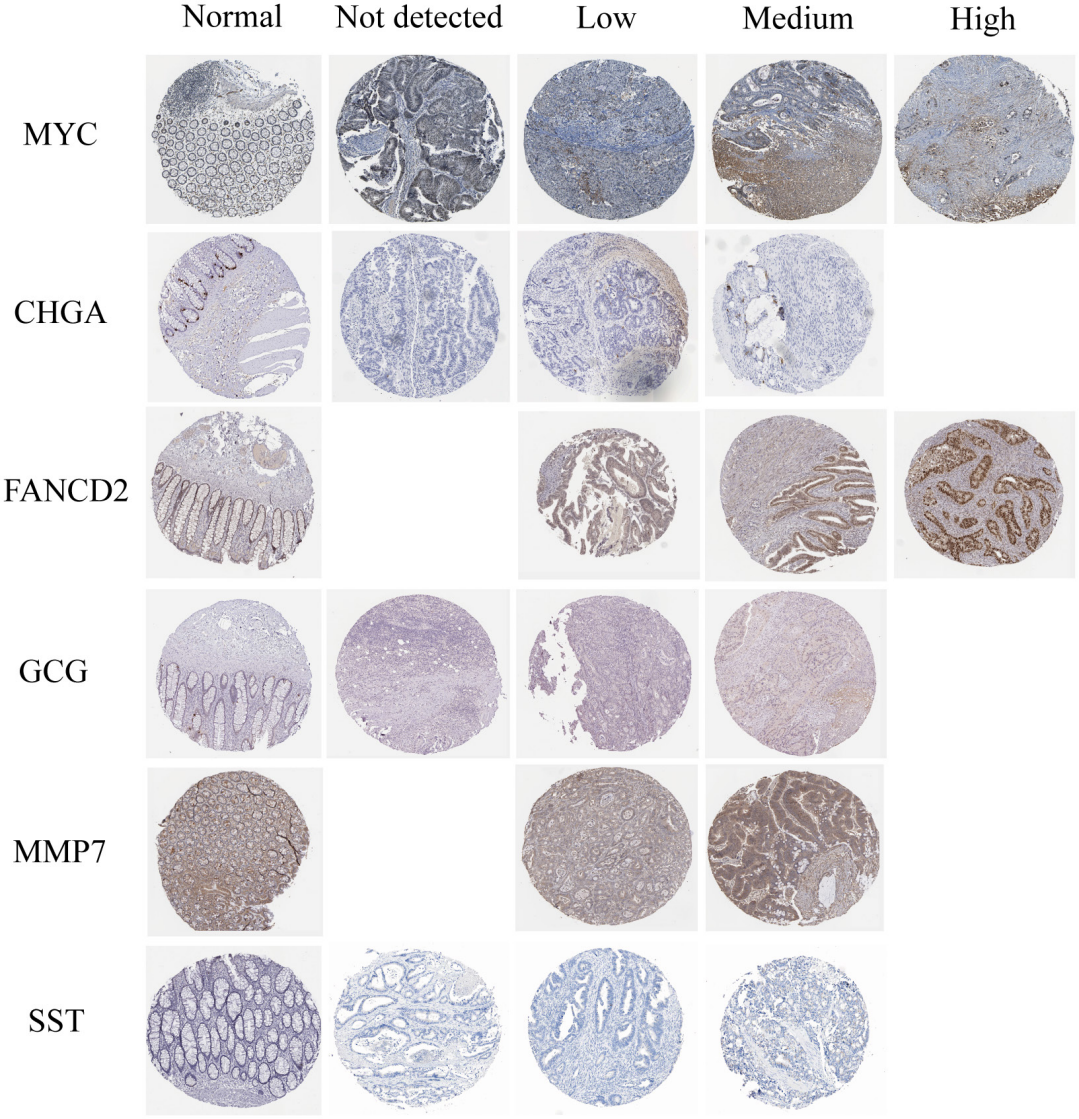
The protein expression levels of the core hub in rectal tissues and colorectal cancer.

The IHC results of the core hub gene protein expression in the HPA database are shown in Figure 8. MYC, GCG, MMP7 and SST are expressed at moderate levels in normal colorectal tissue. CHGA and FANCD2 are expressed at high levels in normal colorectal tissue. The core hub genes also exhibit varied expression levels in colorectal cancer patients, ranging from undetectable low expression to moderate‐high expression. The different expression levels of hub genes may explain the survival differences in colorectal cancer patients.

### 3.9 Model construction

**FIGURE 9:**
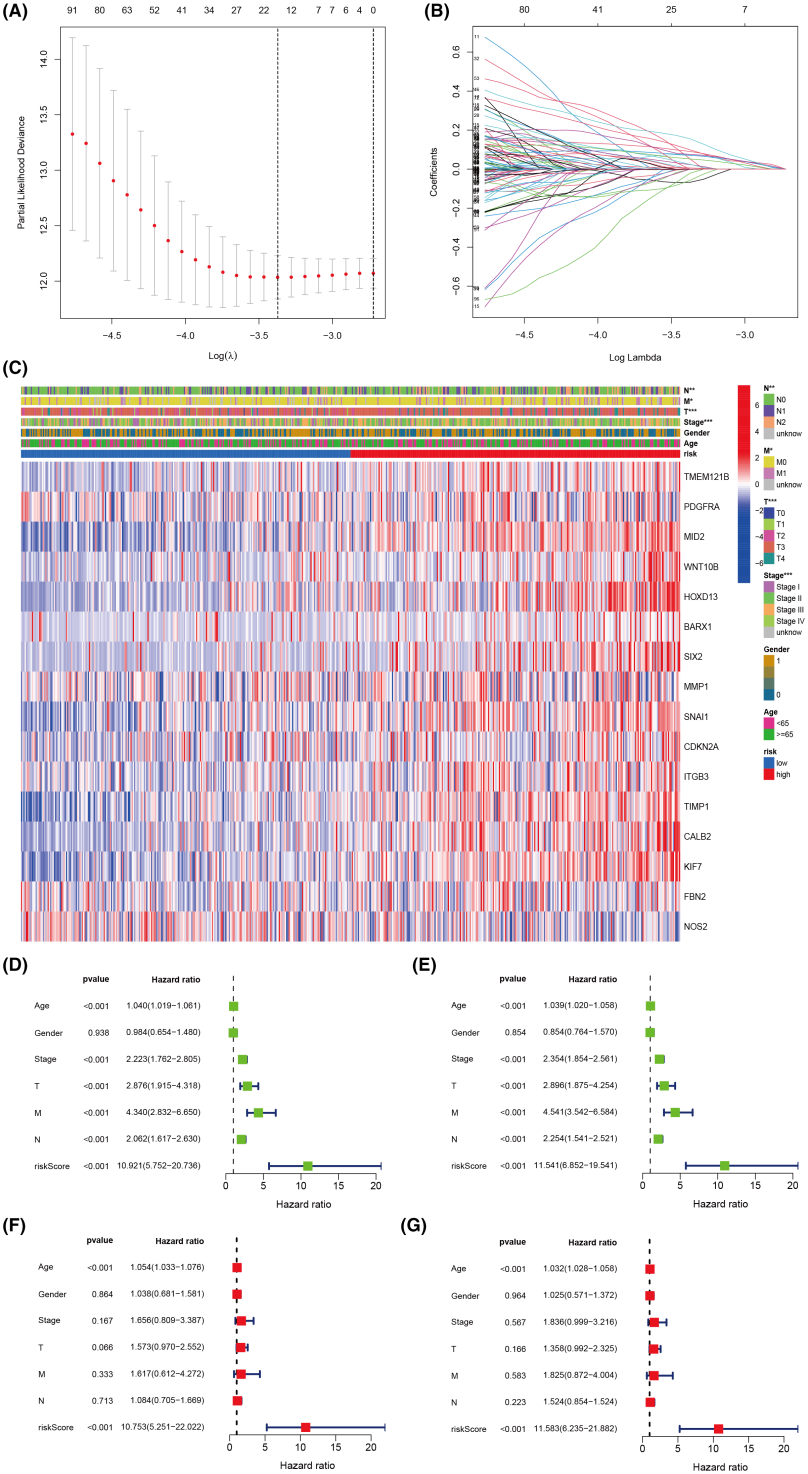
Model Construction (A) Prognostic Lasso Coefficient Selection. (B) Prognostic Lasso Variable Trajectory. (C) Model Gene Heatmap. (D) Univariate Analysis of the Training Set. (E) Univariate Analysis of the Test Set. (F) Multivariable Analysis of the Training Set. (G) Multivariable Analysis of the Test Set.

When using the Lasso regression model for predictive modelling, choose the optimal coefficients. When using the Lasso model with multiple iterations or different parameter settings, the coefficient trajectories of each predictor variable (feature) are shown (Figure 9A,B). The heatmap of gene expression data shows elevated expression of related genes such as *TMEM121B*, *PDGFRA*, *MID2*, *WNT10B*, *HOXD13*, *BARX1*, *SIX2*, *MMP1*, *SNAL1*, *CDKN2A*, *ITGB3*, *TIMP1* and *CALB2* in the high‐risk group (Figure 9C). Univariate analysis of the training set shows Age Hazard ratio = 1.04, Stage Hazard ratio = 2.22, T Hazard ratio = 2.87, M Hazard ratio = 4.34, Riskscore Hazard ratio = 10.92 (Figure 9D). Analysis of the test set shows Riskscore Hazard ratio = 11.54 (Figure 9E). Multivariate analysis of the training set shows Age Hazard ratio = 1.05, Riskscore Hazard ratio = 10.75 (Figure 9F). Multivariate analysis of the test set shows Age Hazard ratio = 1.03, Riskscore Hazard ratio = 11.58 (Figure 9G).

### 3.10 Model validation

**FIGURE 10:**
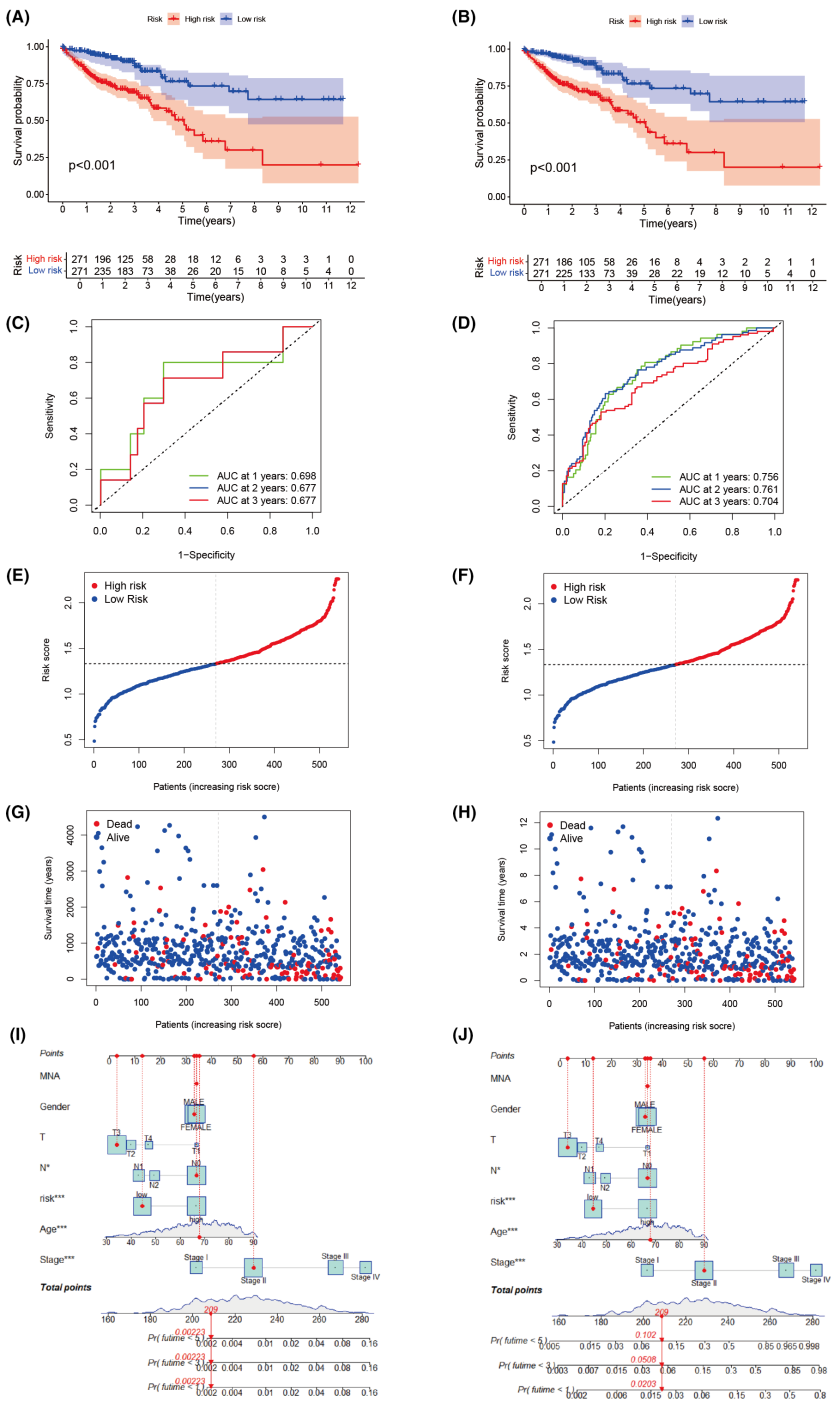
Model construction and evaluation. (A, B) Kaplan–Meier curves are used for training, verification of survival state and survival time. (C, D) Receiver pperating characteristic (ROC) curves show the potential of the predictive model to predict 1‐, 2‐ and 3‐year overall survival (OS) in the training and validation groups. (E–H) Distribution of risk scores and the distribution of overall survival status and risk scores in the training group and the validation group. (J–L) Training, verification of the nomogram display.

For the constructed model, samples are divided into training and testing groups to validate the clinical significance of the model's predictions. Survival curves indicate the presence of survival differences between high and low scoring groups, with statistical significance (Figure 10A,B). Furthermore, in predicting survival years, both the training and test sets demonstrate the model's ability to predict survival rates at 1 year, 2 years and 3 years (Figure 10C,D). Visualization of the distribution of risk scores and OS status indicates the rational distribution of samples in the two risk groups (Figure 10E–H). Additionally, schematic line graphs of the model for the training and test sets are presented (Figure 10I,J).

### 3.11 Functional analysis

**FIGURE 11:**
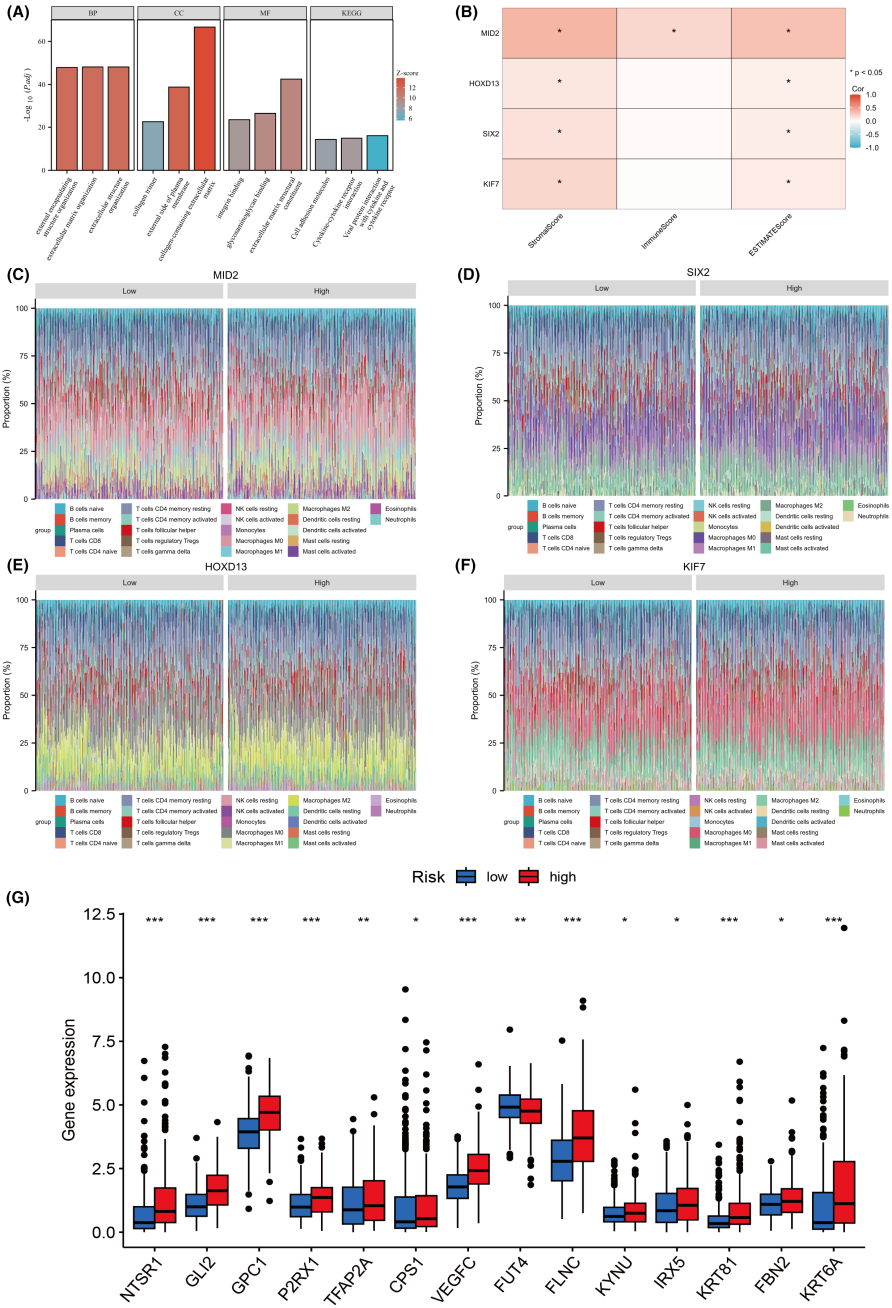
Functional Analysis. (A) Enrichment analysis of differential genes. (B) Tumour microenvironment score of core genes. (C) Overlay column chart of immune infiltration for *MID2*. (D) Overlay column chart of immune infiltration for *SIX2*. (E) Overlay column chart of immune infiltration for *HOXD13*. (F) Overlay column chart of immune infiltration for *KIF7*. (G) Checkpoint differential plot. *p < 0.05; **p < 0.01; ***p < 0.001.

The function primarily enriches the relevant pathways of tumour differentiation and apoptosis (Figure 11A). Calculate the immune infiltration score, stromal score and estimate score of the samples, and plot a heatmap (Figure 11B). The results of the estimate algorithm indicate high scores for stem cells and stromal infiltration. Based on the analysis of public data on the differences in immune infiltration results between groups with high and low expression of molecules (medians), use the CIBERSORT core algorithm to display the data distribution with overlaid bar graphs. The overlaid bar graphs show that *MID2*, *HOXD13*, *SIX2* and *KIF7* are associated with various immune cells, involving complex immune regulatory mechanisms (Figure 11C–F). The high and low‐risk groups have multiple genes related to checkpoint sites, indicating an association with checkpoint sites (Figure 11G).

### 3.12 Constructing a diagnostic model based on the intersection genes of anal fistula and colorectal cancer

**FIGURE 12:**
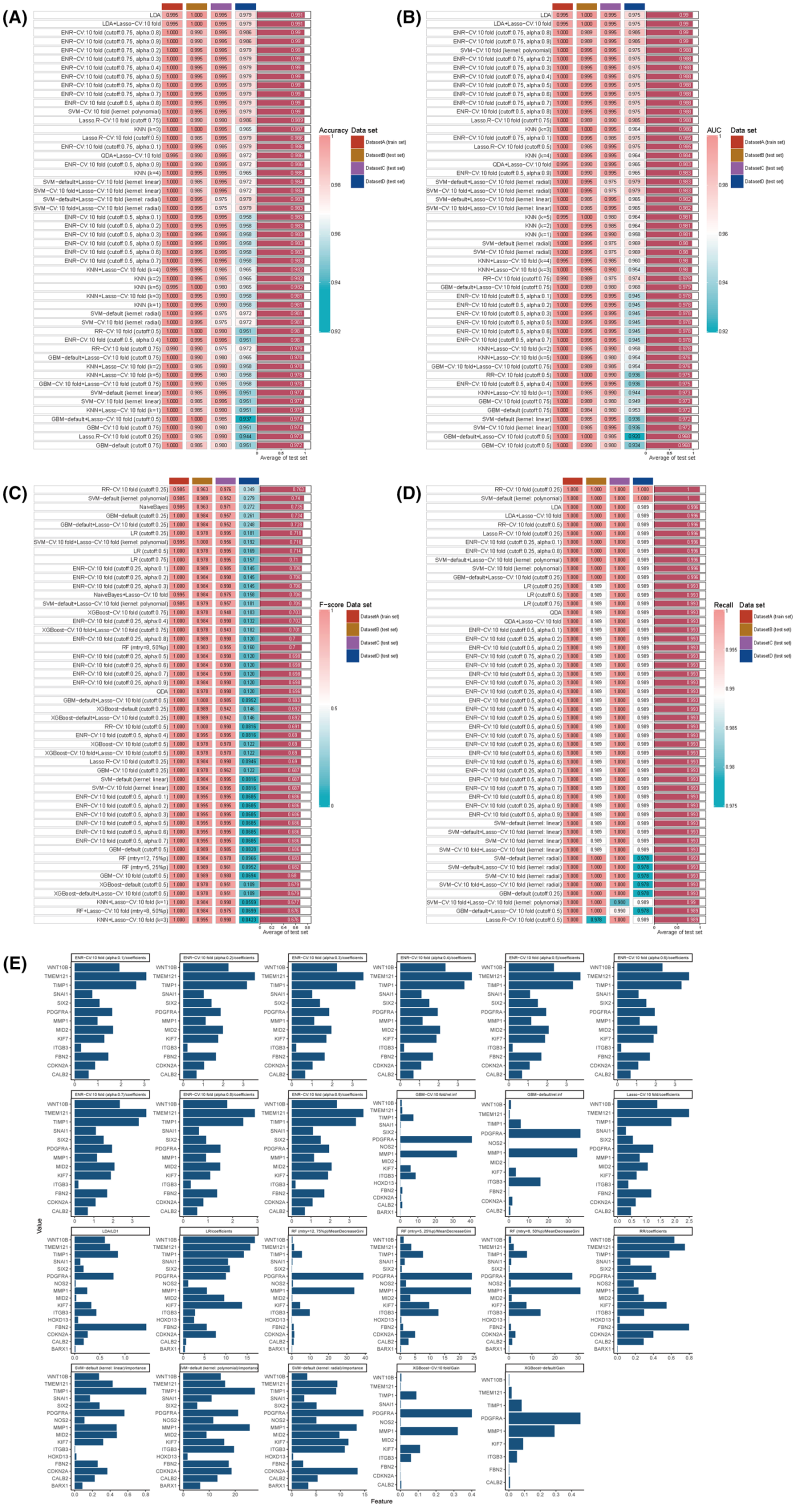
Machine learning builds predictive models. (A) Validation of 207 prediction models through 15 machine learning re‐cross validation frameworks and further calculation of a partial presentation of the C‐index for each model on all datasets. (B) Validation of 207 prediction models through 15 machine learning re‐cross validation frameworks and further calculation of a partial presentation of the AUC index for each model across all datasets. (C) Verifies 207 prediction models through 15 machine learning re‐cross validation frameworks, and further calculates a partial presentation of the F‐index for each model on all datasets. (D) Validation of 207 prediction models through 15 machine learning re‐cross validation frameworks and further calculation of the recall rate of each model on all datasets. (E) Display of genetic composition of the dominant model.

In this study, we developed a diagnostic model using the overlapping genes between anal fistula and colorectal cancer. To achieve this, we employed a hybrid approach involving 15 machine learning algorithms for analysing the common genes associated with these conditions. The colorectal cancer dataset was partitioned into a training set (Dataset A), testing set 1 (Dataset B), testing set 2 (Dataset C) and an internal validation set (Dataset D) randomly sampled from the first three sets in a 1:1:1 ratio. In the training set, we fitted 101 predictive models using a 10‐fold cross‐validation framework, and calculated the C‐index, area under the ROC curve, F‐score, and recall for all training and validation sets, as shown in Figure 12A–D. After comprehensive consideration, the SVM‐default (kernel: linear) model was selected. Consequently, the genes *WNT10B*, *TMEM121B*, *TIMP1*, *SNAI1*, *SIX2*, *PDGFRA*, *NOS2*, *MMP1*, *MID2*, *KIF7*, *ITGB3*, *HOXD13*, *FBN2*, *CDKN2A*, *CALB2* and *BARX1* were chosen (Figure 11E).

### 3.13 Mendelian randomization

**FIGURE 13:**
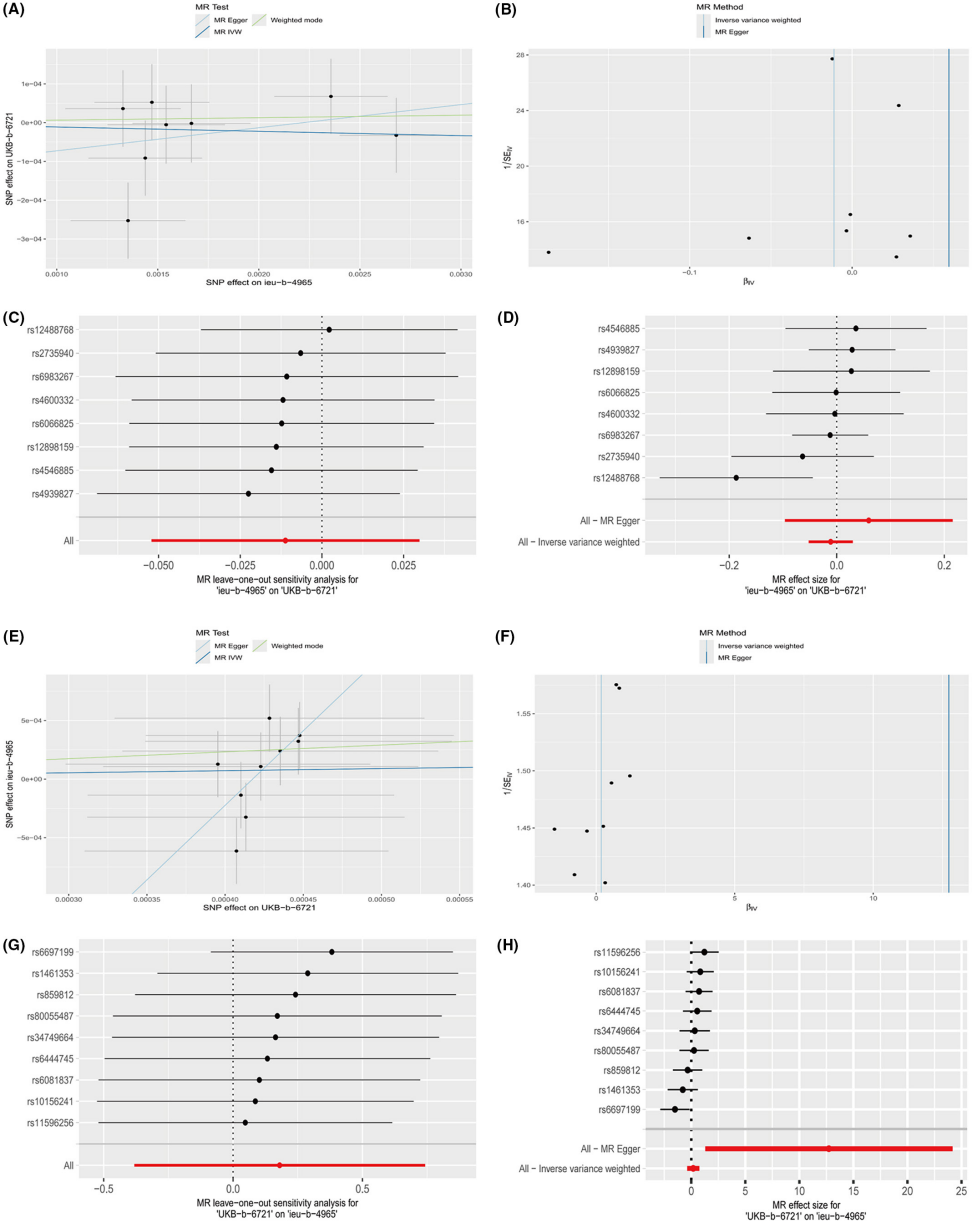
Mendelian randomization (MR). (A) Scatterplot of IEU‐b‐4965||UKB‐b‐6721. (B) Funnel plot of IEU‐b‐4965||UKB‐b‐6721. (C) Leave‐one‐out method of IEU‐b‐4965||UKB‐b‐6721. (D) SNP forest plot of IEU‐b‐4965||UKB‐b‐6721. (E) Scatter plot of UKB‐b‐6721||IEU‐b‐4965. (F) Funnel plot of UKB‐b‐6721||IEU‐b‐4965. (G) Leave‐one‐out method of UKB‐b‐6721||IEU‐b‐4965. (H) SNP forest plot of UKB‐b‐6721||IEU‐b‐4965.

By utilizing two‐sample MR and bidirectional MR, the relationship between anal fistula and colorectal cancer was further explored. The MR results indicated no causal relationship between anal fistula and colorectal cancer (Table 1; Figure 13A,E). The forest plot of SNPs further confirmed the results (Figure 13D,H). Results from the funnel plot and leave‐one‐out analysis showed that pleiotropy and heterogeneity did not affect the outcomes (Figure 13B,C,F,G).

**TABLE 1:** The Mendelian randomization (MR) results indicated no causal relationship between anal fistula and colorectal cancer.

id. exposure	id. outcome	Outcome	Exposure	Results from two sample MR										Heterogeneity tests				Test for directional horizontal pleiotropy			Test that the exposure is upstream of the outcome	
				Method	nsnp	b	se	*p* value	lo_ci	up_ci	or	or_lci95	or_uci95	Method	Q	Q_df	Q_*p* value	Egger_intercept	se	*p* value	Correct_causal_direction	Steiger_*p* value
ieu‐b‐4965	UKB‐b‐6721	Diagnoses—main ICD10: K60.3 Anal fistula	Colorectal cancer	MR Egger	8	0.060	0.079	0.482	−0.096	0.215	1.061	0.908	1.240	MR Egger	7.200	6	0.303					
ieu‐b‐4965	UKB‐b‐6721	Diagnoses—main ICD10: K60.3 Anal fistula	Colorectal cancer	MR IVW	8	−0.011	0.021	0.593	−0.052	0.030	0.989	0.949	1.030	Inverse variance weighted	8.220	7	0.313	0.000	0.000	0.392	TRUE	0.655
ieu‐b‐4965	UKB‐b‐6721	Diagnoses—main ICD10: K60.3 Anal fistula	Colorectal cancer	Weighted mode	8	0.006	0.030	0.835	−0.052	0.064	1.006	0.950	1.067									
UKB‐b‐6721	ieu‐b‐4965	Colorectal cancer	Diagnoses—main ICD10: K60.3 Anal fistula	MR Egger	9	12.731	5.836	0.065	1.293	24.170	338175.088	3.644	31387065473.761	MR Egger	7.780	7	0.353					
UKB‐b‐6721	ieu‐b‐4965	Colorectal cancer	Diagnoses—main ICD10: K60.3 Anal fistula	MR IVW	9	0.180	0.287	0.530	−0.382	0.742	1.197	0.683	2.100	Inverse variance weighted	12.920	8	0.114	−0.005	0.002	0.068	TRUE	0.865

## 4 DISCUSSION

This study explores the potential correlation and molecular mechanisms between anal fistula and colorectal cancer through a comprehensive analysis of gene microarray data, clinical information and multiple bioinformatics databases. Thirteen core genes were identified, contributing to essential biological processes like extracellular matrix functions, signalling transduction, cell growth and protein modification. These genes are closely linked to key pathways such as the Wnt signalling pathway and other pathways associated with colorectal cancer. The findings align with previous research, including the significant role of the Wnt signalling pathway in colorectal cancer progression and the crucial role of the extracellular matrix in the tumour microenvironment.^23^, ^24^

Thirteen core genes have been identified to play crucial roles in various biological processes essential for tumour development and progression. The involvement of these genes in extracellular matrix functions, signalling transduction, cell growth and protein modification underscores their significance in maintaining the tumour microenvironment and promoting tumour growth. The study highlights the Wnt signalling pathway as a well‐known driver of colorectal cancer development. Aberrant activation of this pathway can lead to uncontrolled cell proliferation and tumour formation, further reinforcing the critical role of the identified core genes associated with this pathway in colorectal cancer. Additionally, two genes, CDKN2A and TIMP1, stand out for their extensive study in colorectal cancer. CDKN2A, also referred to as p16, acts as a tumour suppressor gene that regulates cell cycle progression. Decreased expression or loss of function of CDKN2A is frequently observed in colorectal cancer and correlates with poor prognosis. On the other hand, TIMP1 serves as an inhibitor of matrix metalloproteinases involved in tissue remodelling and tumour invasion. Reduced TIMP1 levels have been linked to heightened tumour aggressiveness and poor survival among colorectal cancer patients.

The analysis of immune infiltration offers further insights into the potential of immunotherapy in colorectal cancer treatment. The findings suggest that the tumour microenvironment in colorectal cancer patients might be conducive to immune‐based therapeutic strategies, aligning with the prevailing trend in immunotherapy research, which focuses on personalized and targeted approaches to leverage the immune system against cancer. The alignment of genes predicted by the diagnostic model with those in the prognosis model underscores the significance of these core genes in both diagnosing and managing colorectal cancer. Notably, the presence of the PDGFRA gene, associated with tumour angiogenesis, hints at the viability of targeting angiogenesis as a potential treatment strategy for colorectal cancer. Similarly, the presence of the MMP1 gene, responsible for encoding matrix metalloproteinase 1, suggests that inhibiting tumour invasion and metastasis could be critical in improving patient outcomes.

The roles of core genes in colorectal cancer prognosis and immune response were further confirmed through prognosis‐related analysis and immune infiltration analysis. In particular, genes CDKN2A and TIMP1 have been shown to be closely related to the prognosis of colorectal cancer.^25^, ^26^ Additionally, the results of immune infiltration analysis suggest the potential value of immunotherapy in the treatment of colorectal cancer, aligning with current trends in immunotherapy research.^27^

The genes predicted by the diagnostic model of the machine learning model are completely consistent with the genes in the constructed prognosis model, emphasizing the important value of these core genes in the diagnosis and differentiation of colorectal cancer. This consistency suggests that these genes may play crucial roles in the pathophysiological processes of colorectal cancer. For example, the PDGFRA gene is related to tumour angiogenesis, which is a key factor in tumour growth and metastasis.^28^ The matrix metalloproteinase 1 encoded by the MMP1 gene plays a role in tumour invasion and metastasis.^29^ The expression levels of these genes may influence the biological behaviour of tumours and thus impact prognosis.

However, despite finding some common core genes and biological processes between anal fistula and colorectal cancer, no causal relationship was found between the two through MR analysis. This indicates that the association between the two may be more influenced by other potential factors. Furthermore, the beta values of the MR results cross zero, which may be due to insufficient sample size or other biases, emphasizing the need for caution in interpreting the results and highlighting the necessity for further research.^30^

## 5 CONCLUSIONS

In conclusion, this study provides preliminary evidence of the correlation between anal fistula and colorectal cancer and reveals possible molecular mechanisms. Future research needs to further explore the association between anal fistula and colorectal cancer, as well as the specific roles of these core genes in disease development. More samples and in‐depth mechanistic studies are needed to validate the results of MR analysis and explore the impact of other possible biological processes and environmental factors on this association.

## AUTHOR CONTRIBUTIONS

**Yicheng Liu:** Conceptualization (equal); resources (equal). **Wenjun Zhao:** Conceptualization (equal); data curation (equal); resources (equal). **Weiye Hu:** Conceptualization (equal); data curation (equal); resources (equal). **Jin Xu:** Formal analysis (equal); software (equal). **Haiyan Zhang:** Data curation (equal); formal analysis (equal); visualization (equal). **Ting Huang:** Data curation (equal); software (equal). **Chuang Wu:** Formal analysis (equal); validation (equal). **Jiajia Yang:** Formal analysis (equal); software (equal). **Wenjing Mao:** Formal analysis (equal); resources (equal); software (equal). **Xiaobing Yao:** Data curation (equal); formal analysis (equal); software (equal). **Yafeng Lu:** Formal analysis (equal); software (equal). **Qingming Wang:** Data curation (equal); supervision (equal); validation (equal).

## FUNDING INFORMATION

This study is sponsored 2023 Shanghai Science and Technology Innovation Action Plan‐Medical Innovation Research Project (23Y11921800) and ‘14th Five‐Year Plan’ Traditional Chinese Medicine Specialty and Traditional Chinese Medicine Emergency Capacity Improvement Project (ZYTSZK1‐8).

## CONFLICT OF INTEREST STATEMENT

The authors declare that the research was conducted in the absence of any commercial or financial relationships that could be construed as a potential conflict of interest.

## Data Availability

All data can be obtained by contacting the corresponding author.
